# Impact of dyslipidemia on the cumulative pregnancy outcomes after first ovarian stimulation

**DOI:** 10.3389/fendo.2022.915424

**Published:** 2022-08-09

**Authors:** Xue Jiang, Xinle Lu, Mingshu Cai, Yu Liu, Yihong Guo

**Affiliations:** ^1^ Center of Reproductive Medicine, The First Affiliated Hospital of Zhengzhou University, Zhengzhou, China; ^2^ Henan Key Laboratory of Reproduction and Genetics, Zhengzhou, China

**Keywords:** dyslipidemia, lipid metabolism, cumulative live birth rate, assisted reproductive technology, polycystic ovary syndrome

## Abstract

**Objectives:**

To investigate the cumulative live birth rate (CLBR) according to lipid metabolism in patients with or without polycystic ovarian syndrome (PCOS) undergoing their first complete *in vitro* fertilization/intracytoplasmic sperm injection (IVF/ICSI) cycles.

**Patients:**

A total of 1,470 patients with PCOS and 3,232 patients without PCOS who underwent their first complete IVF/ICSI cycles from January 2016 to June 2018 were included. During a minimum of 2 years of follow-up, they had achieved at least one live birth or used all available embryos. The cumulative pregnancy outcomes were compared based on the patients’ blood lipid parameters, including triglycerides (TG), total cholesterol (TC), low-density lipoprotein cholesterol (LDL-C) and high-density lipoprotein cholesterol (HDL-C), in the two populations. Patients with an abnormal level of one or more of these four indicators were considered the dyslipidemia group. Patients whose four indicators were normal were considered the control group.

**Results:**

Among 1,470 patients with PCOS, the cumulative pregnancy outcomes were similar in the dyslipidemia group and control group. Logistic regression analysis showed that the TC levels were significantly negatively associated with the cumulative live birth rate (CLBR) after adjustment for confounding factors such as age and BMI (aOR 0.81, 95% CI 0.66-0.98, P<0.05). Among the 3,232 patients without PCOS, there was no significant difference in the cumulative pregnancy outcomes between the dyslipidemia group and the control group. No significant correlations were found in other logistic regression analyses.

**Conclusions:**

TC negatively impacts the CLBR after first ovarian stimulation in PCOS patients. PCOS patients with dyslipidemia caused by elevated TC may have a poor CLBR.

## Introduction

Polycystic ovary syndrome (PCOS) is a common endocrinopathy in women of reproductive age characterized by anovulation, hyperandrogenemia and polycystic ovaries (1). PCOS is also the main factor causing anovulatory infertility in child-bearing period female (2). Assisted reproductive technology (ART) is widely used for infertility treatment of PCOS patients. Due to their characteristics such as hyperandrogenemia, abnormal LH levels or insulin resistance, COS patients have the characteristics of a higher ovarian response, more oocytes retrieved, a larger number of transferable embryos, a lower fertilization rate, a higher incidence of ovarian hyperstimulation syndrome (OHSS) and pregnancy complications during the treatment process (3, 4). PCOS is usually accompanied by metabolic disorders such as insulin resistance, obesity, dyslipidemia and thyroid dysfunction (5, 6). It is also a high-risk factor for cardiovascular disease, type 2 diabetes and endometrial cancer (7, 8). Previous studies have focused on the effect of obesity on pregnancy outcomes in PCOS patients. One study showed that PCOS patients with obesity had a considerably higher miscarriage risk and lower clinical pregnancy rate (9). Since an adequate number of oocytes per ovarian stimulation can be retrieved from most PCOS patients, considerable embryos can be provided for fresh transfer or subsequent frozen–thawed embryo transfer (FET). Therefore, the cumulative live birth rate (CLBR) is actually a better indicator for estimating the treatment outcomes of infertile patients with PCOS (10, 11) and has recently been strongly recommended as a suitable measure of ART success. Our previous study showed that obesity had a negative impact on the cumulative clinical pregnancy rate (CCPR) and CLBR (12, 13). Dyslipidemia is frequently accompanied by obesity and is common in PCOS (14–16). Dyslipidemia may present with abnormal or more types of blood lipids, including elevated total cholesterol (TC), triglycerides (TG), and low-density lipoprotein cholesterol (LDL-C) concentrations, as well as reduced high-density lipoprotein cholesterol (HDL-C) levels (17). A recent study showed that abnormal lipid metabolic parameters had an adverse effect on the CLBR following IVF/ICSI among patients without PCOS (18). Another study showed that dyslipidemia was associated with a greater number of oocytes retrieved in PCOS patients who underwent an unstimulated cycle (19). However, relation of dyslipidemia with cumulative pregnancy outcomes in PCOS patients is not known. Therefore, we performed the current study to evaluate the impact of dyslipidemia on cumulative pregnancy outcomes after the first ovarian stimulation.

## Materials and methods

### Study subjects and design

This was a retrospective cohort study at the Reproductive Medicine Center of the First Affiliated Hospital of Zhengzhou University. We included 1,470 patients diagnosed with PCOS by the Rotterdam criteria (20) and 3,232 patients with tubal factor infertility as the reference population. These infertile patients underwent their first IVF/ICSI cycle from January 2016 to June 2018 and achieved at least one live birth or used all fresh and frozen embryos during a follow-up period of at least 2 years. The data for this cohort study were retrieved from the Clinical Reproductive Medicine Management System/Electronic Medical Record Cohort Database (CCRM/EMRCD) at the Reproductive Medical Center, First Affiliated Hospital of Zhengzhou University. This study received approval from the Institutional Review Board and Ethics Committee of the First Affiliated Hospital of Zhengzhou University. All patients signed written informed consent forms.

Cycles with the following conditions were excluded: (i) one or both members of the couple had abnormal karyotypes; (ii) cycles utilized donor oocytes or sperm; (iii) preimplantation genetic testing for aneuploidy (PGT-A) cycles, preimplantation genetic testing for monogenic/single gene defect (PGT-M)cycles, and preimplantation genetic testing for chromosomal structural rearrangement (PGT-SR) cycles; (iv) cycles with no viable embryos; (v) patients with other endocrine/metabolic diseases; (vi) patients with recurrent spontaneous abortion; (vii) patients who discontinued fertility treatment for personal reasons with remaining embryos; (viii) cycles with missing data or loss to follow-up.

Serum biochemical parameters, including TG, TC, LDL-C and HDL-C, were measured in a single laboratory before ovarian stimulation. According to the 2016 Chinese guidelines for the management of dyslipidemia in adults (17), patients were defined as having dyslipidemia if any one of the following abnormal indicators was present: TC ≥ 6.22 mmol/L (240 mg/dl), LDL-C ≥ 4.14 mmol/L (160 mg/dl), HDL-C ≤ 1.04 mmol/L (40 mg/dl), or TG ≥ 2.26 mmol/L (200 mg/dl). Those without the above conditions were regarded as the normal group. We investigated the effect of dyslipidemia on the CLBR in the two populations.

### IVF/ICSI protocol

The patients included in the study were treated with gonadotropin-releasing hormone (GnRH) agonists for pituitary desensitization to prevent a premature surge in luteinizing hormone. According to the patient’s characteristics, a suitable ovarian stimulation protocol was formulated for each person by the medical experts. Follicular phase protocol: Patients were intramuscularly injected with triptorelin depot (decapeptyl 3.75 mg; Ipsen Pharma, France) on days 2-3 of the menstrual cycle. After 28 to 42 days, pituitary downregulation was achieved (E2 < 50 pg/ml, LH < 3 mIU/ml and ovary cysts less than 10 mm). Luteal phase protocol: Intramuscular administration of triptolide (Ferring GmbH, 0.1 mg, Switzerland; Ipsen Pharma Biotech, 0.1 mg, France) was performed in the mid-luteal phase, and the dose was reduced to 0.05 mg/d after 10 days until pituitary downregulation was achieved. Follicle-stimulating hormone (FSH) was administered to stimulate follicle growth (Gonal-F, Serono, Puregon, Netherlands, u-FSH, Livzon), and the initial dose was dependent on the patient’s characteristics and the antral follicle count (AFC). Experienced clinicians regularly monitored follicle development by transvaginal ultrasound and the serum estradiol, progesterone and LH levels, and the subsequent dose was adjusted according to these indicators. If necessary, human menopausal gonadotropin (HMG, Livzon) can be added. Human chorionic gonadotropin (hCG, Livzon) and recombinant human chorionic gonadotropin (Merck Serono, Italy) are used to trigger oocyte maturation when the maximal follicle diameter is greater than 20 mm and more than 2/3 of the follicles reach 16 mm in diameter. Transvaginal ultrasound-guided ovum pick-up (OPU) was performed 36-37 hours after hCG administration. Progesterone vaginal gel (Merck Serono, Switzerland) was used for luteal support from the day of OPU. The insemination method was chosen according to the sperm parameters and fertilization situation. After laboratory culture, the patients underwent cleavage-stage embryo or blastocyst transfer according to the development of the embryos. For patients who were not suitable for fresh embryo transfer due to OHSS, insufficient endometrial thickness and abnormal laboratory parameters, the whole embryo freezing strategy was adopted. Endometrial preparation schemes for subsequent FET included natural cycles and artificial cycles using estradiol.

### Main outcomes measures

The primary outcome of our study was the CLBR, and the secondary outcome was the CCPR. The CLBR was defined as at least one live infant born after 24 weeks of gestation in the fresh transfer cycles or subsequent FET cycles. Only the first live birth was included in the analysis. The CCPR was calculated based on the detection of intrauterine gestational sacs *via* ultrasonography after embryo transfer (10).

### Statistical analysis

Statistical analyses were conducted using IBM SPSS Statistics for Mac (IBM Corp., Armonk, NY, USA), version 26.0. Continuous variables with a normal distribution are presented as the means ± standard deviations (SDs), and differences between groups were compared by Student’s t test. Continuous variables with skewed distributions are presented as medians (P25-P75) and were assessed using the Mann–Whitney U test. Categorical variables are expressed as frequencies (percentages) and were compared using the chi-square test. Logistic regression analysis was used to determine the influence of dyslipidemia on cumulative pregnancy outcomes. The results are expressed by the adjusted odds ratio (aOR) and 95% confidence interval (CI). All tests were two-sided, and *P*<0.05 was considered statistically significant.

## Results

**Figure 1:**
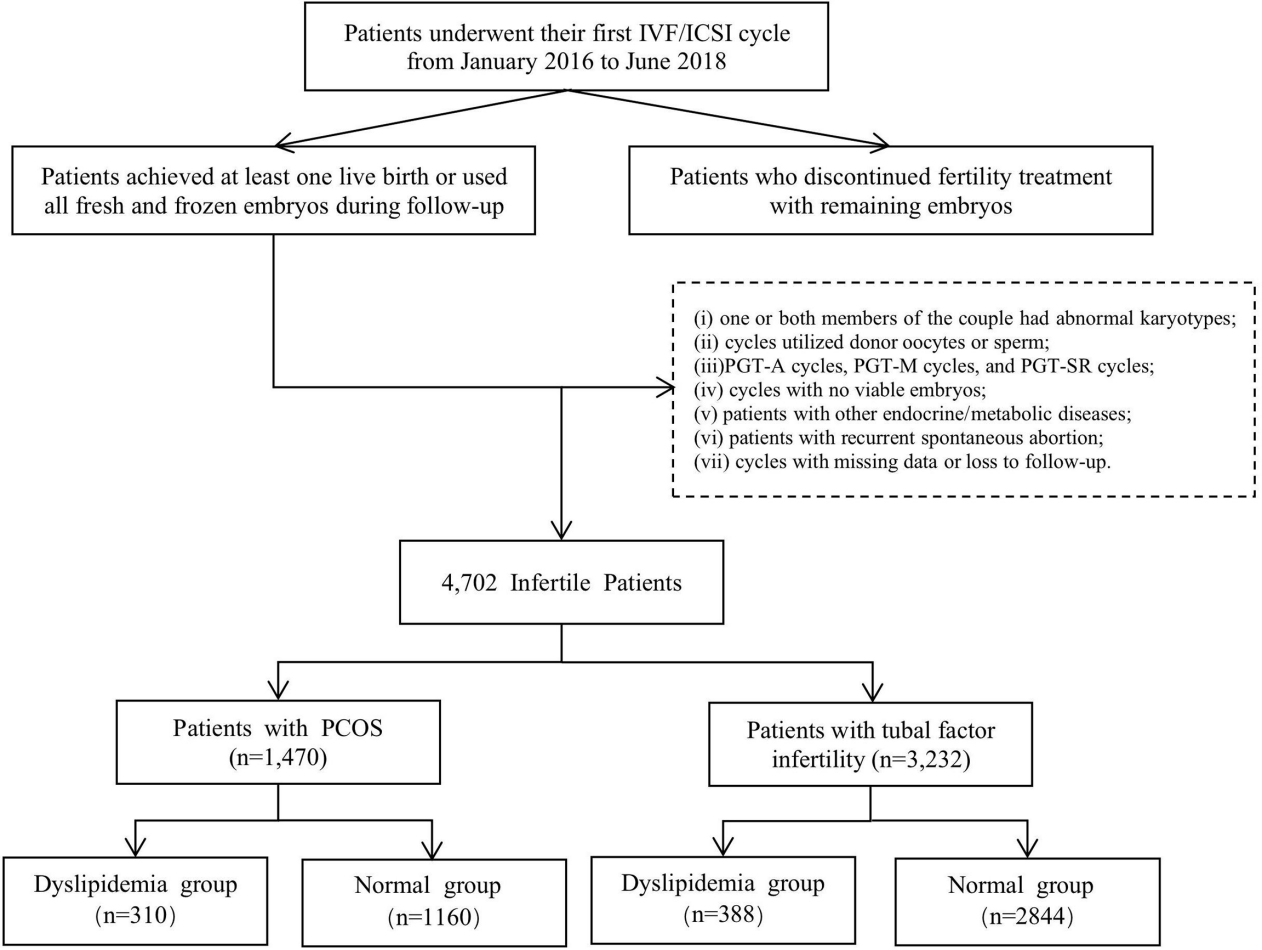
Flowchart of patient selection.

In total, 1,470 patients with PCOS and 3,232 patients without PCOS underwent a complete ART cycle after the first ovarian stimulation (**Figure 1**). A complete ART cycle was defined as achieving at least one live birth with or without embryos remaining afterward or as not achieving a live birth after using all viable embryos from ovarian stimulation. The comparison of body mass index (BMI) and lipid parameters between the two populations is shown in **Table 1**. A total of 310 (21.1%) PCOS patients were diagnosed with dyslipidemia, while 388 (12.0%) non-PCOS patients were diagnosed with dyslipidemia. The difference between the two groups was statistically significant (*P*<0.05). Compared with patients with tubal factor infertility, PCOS patients showed increased BMI (22.7 ± 3.0 vs. 24.0 ± 3.6,kg/m^2^), elevated TG levels (1.1 ± 4.3vs. 1.4 ± 0.9,mmol/L) and reduced HDL-C (1.5 ± 0.3 vs. 1.4 ± 0.4,mmol/L) (*P*<0.05) concentrations. The incidence of BMI ≥ 24 kg/m^2^(49.7% vs. 30.9%), hypertriglyceridemia (TG ≥ 2.26 mmol/L, 12.1% vs. 3.9%) and HDL-C hypolipidemia (HDL-C ≤ 1.04 mmol/L, 12.7% vs. 8.6%) was also significantly higher than those of the non-PCOS group. Therefore, we analyzed the relationship between blood lipid metabolism and cumulative pregnancy outcomes in both groups.

**Table 1:** Comparison of lipid metabolism characteristics between PCOS patients and non-PCOS patients.

Variable	Patients for tubal factor (*N*=3232)	Patients with PCOS (*N*=1470)	*P* value
BMI (kg/m^2^)	22.7±3.0	24.0±3.6	<0.001
<24	2229 (69.1)	739 (50.3)	<0.001
≥24	995 (30.9)	730 (49.7)	
Dyslipidemia, n (%)	388 (12.0)	310 (21.1)	<0.001
Lipid Metabolic Parameters			
TG (mmol/L)	1.1±4.3	1.4±0.9	0.027
≥2.26	125 (3.9)	178 (12.1)	<0.001
TC (mmol/L)	4.2±0.8	4.3±0.7	NS
≥6.22	18 (0.6)	15 (1.0)	NS
HDL-C (mmol/L)	1.5±0.3	1.4±0.4	<0.001
≤1.04	278 (8.6)	186 (12.7)	<0.001
LDL-C (mmol/L)	2.5±0.7	2.5±1.1	NS
≥4.14	326 (10.1)	158 (10.7)	NS

Data are presented as the mean±SD or frequency (percentage).BMI, Body mass index; TC, =total cholesterol; TG, triglyceride; LDL-C, low-density lipoprotein cholesterol; HDL-C, high density lipoprotein cholesterol. NS, not significant.

**Table 2** shows the clinical and ART characteristics of the PCOS patients in terms of the dyslipidemia and non-dyslipidemia groups. Patients with normal blood lipid metabolism were used as the reference group. The mean age of the dyslipidemia group was slightly higher than that of the reference group (28.8 ± 3.6 vs. 28.3 ± 3.6, years *P*<0.05). The average BMI of the dyslipidemia group was significantly higher than that of the reference group (25.7 ± 3.0 vs. 23.6 ± 3.6,kg/m^2^), and the proportion of patients with BMI ≥ 24 kg/m^2^ was significantly higher (71.3% vs. 43.9%) (*P*<0.05). There was no significant difference in male age or BMI between the two groups (*P*>0.05). Regarding the endocrine and metabolic indexes, the dyslipidemia group showed higher levels of TG, TC and LDL-C and lower HDL-C concentrations, as mentioned above (*P*<0.05). The two groups were comparable in terms of basal FSH, basal LH and Anti-Müllerian hormone (AMH) levels (*P*>0.05). The fasting blood glucose of the dyslipidemia group was slightly higher than that of the reference group (*P*<0.05), whereas it was within the normal range. In terms of ART characteristics, the administered total gonadotropin dose in the dyslipidemia group was greater than that in the reference group (*P*<0.05). Additionally, there were no significant differences in the parameters of oocytes and embryos, insemination methods or the incidence of OHSS (*P*>0.05). The differences in the cumulative pregnancy outcomes between the two groups were not statistically significant (dyslipidemia group vs. reference group, CCPR: 90.6% vs. 89.5%, CLBR: 83.9% vs. 85.2%, *P>*0.05).

**Table 2:** Comparison of study objects between PCOS patients with and without dyslipidemia.

Characteristics	Dyslipidemia group	Normal group	*P* value
No. of case (*N*=1470)	310 (21.1)	1160 (78.9)	
**Clinical characteristics**			
Age of female (years)	28.8±3.6	28.3±3.6	0.030
<35	288 (92.9)	1094 (94.3)	NS
≥35	22 (7.1)	66 (5.7)	
Age of male (years)	29.0 (26.0-32.0)	29.0 (27.0-32.0)	NS
BMI of female (kg/m^2^)	25.7±3.0	23.6±3.6	<0.001
<24	89 (28.7)	650 (56.1)	<0.001
≥24	221 (71.3)	509 (43.9)	
BMI of male (kg/m^2^)	24.3 (22.3-27.4)	24.8 (22.3-27.3)	NS
Infertility type, n (%)			NS
Primary	215 (69.4)	810 (69.8)	
Secondary	95 (30.6)	350 (30.2)	
Basal FSH (IU/L)	5.4 (4.6-6.3)	5.6 (4.7-6.6)	NS
Basal LH (IU/L)	7.6 (4.3-12.2)	7.6 (4.7-13.4)	NS
AMH (ng/ml)	7.8 (5.1-10.7)	7.5 (5.6-10.7)	NS
Antral Follicle Count (AFC)	24 (24.0-24.0)	24 (24.0-24.0)	NS
FBG (mmol/L)	4.9 (4.5-5.2)	4.8 (4.5-5.1)	0.034
Lipid Metabolic Parameters			
TG (mmol/L)	2.4 (1.5-3.0)	1.0 (0.7-1.4)	<0.001
TC (mmol/L)	4.3 (3.8-5.0)	4.2 (3.8-4.7)	0.014
HDL-C (mmol/L)	1.0 (0.9-1.2)	1.5 (1.3-1.7)	<0.001
LDL-C (mmol/L)	2.7 (2.2-3.2)	2.4 (2.0-2.9)	<0.001
**ART characteristics**			
Total gonadotropin dose (IU)	2400.0 [1871.9-3115.6]	1875.0 [1425.0-2525.0]	<0.001
No. of oocytes retrieved	18.0 [13.0-24.0]	17.0 [12.3-23.0]	NS
Insemination method, n (%)			NS
IVF	257 (82.9)	925 (79.7)	
ICSI	47 (15.2)	210 (18.1)	
IVF+ICSI	6 (1.9)	25 (2.2)	
No. of 2PN embryos	11 [7.0-16.0]	10 [7.0-15.0]	NS
No. of available embryos	5.0 [4.0-8.0]	6.0 [4.0-8.0]	NS
OHSS, n (%)	110 (35.5)	420 (36.2)	NS
CCPR, n (%)	281 (90.6)	1038 (89.5)	NS
CLBR, n (%)	260 (83.9)	988 (85.2)	NS

Data are presented as mean±SD, the median (inter quartile range, 25th-75th) or frequency (percentage). BMI, Body mass index; FSH, folliclestimulating hormone; LH, luteinizing hormone; AMH, anti-Mullerian hormone; AFC, antral follicle count; FBG, Fasting Blood-Glucose; TC, total cholesterol; TG, triglyceride; LDL-C, low-density lipoprotein cholesterol; HDL-C, high density lipoprotein cholesterol; ART, assisted reproductive technology; IVF, in vitro fertilization; ICSI, intracytoplasmic sperm; NS, not significant; OHSS, ovarian hyperstimulation syndrome; CLBR, cumulative live birth rate; CCPR, cumulative clinical pregnancy rate.

**Table 3** shows the comparison of the clinical and ART characteristics between patients with and without dyslipidemia in patients with tubal factor infertility. There was no significant difference in the average age of patients between the two groups or in male age (*P*>0.05). Compared with the reference group, the dyslipidemia group showed a higher mean BMI (22.5 ± 2.9 vs. 24.4 ± 2.9, kg/m^2^) and more patients with BMI ≥ 24 kg/m^2^ (55.3% vs. 27.5%) (*P*<0.05). Baseline serum FSH and LH levels were lower in the dyslipidemia group than in the reference group (*P*<0.05). Similar to the PCOS group, the fasting blood glucose in the dyslipidemia group was slightly higher than that in the control group (*P*<0.05) but within the normal range. In terms of ART characteristics, there was no significant difference in other aspects except for a significantly higher total gonadotropin dose in the dyslipidemia group (*P*<0.05). The cumulative pregnancy outcomes were comparable in the two groups (dyslipidemia group vs. reference group, CCPR: 76.5 vs. 74.8%, CLBR: 69.6% vs. 69.3%, *P>*0.05).

**Table 3:** Comparison of study objects between non-PCOS patients with and without dyslipidemia.

Characteristics	Dyslipidemia group	Normal group	*P* value
No. of case(*N*=3232)	388(12.0)	2844(88.0)	
**Clinical characteristics**			
Age of female(years)	31.9±5.5	31.6±5.3	NS
<35	281(72.4)	2024(71.2)	NS
≥35	107(27.6)	820(28.8)	
Age of male(years)	32.0[28.0-37.0]	31.0[28.0-37.0]	NS
BMI of female(kg/m^2^)	24.4±2.9	22.5±2.9	<0.001
<24	172(44.7)	2057(72.5)	<0.001
≥24	213(55.3)	782(27.5)	
BMI of male(kg/m^2^)	25.2[22.9-27.3]	24.9[22.5-27.4]	NS
Infertility type, n (%)			NS
Primary	127(32.7)	1031(36.3)	
Secondary	261(67.3)	1813(63.7)	
Basal FSH (IU/L)	6.3[5.4-7.5]	6.6[5.6-8.0]	0.002
Basal LH (IU/L)	4.2[3.0-5.6]	4.7[3.6-6.2]	<0.001
AMH(ng/ml)	2.6[1.5-4.3]	2.6[1.4-4.2]	NS
Antral Follicle Count(AFC)	12.0[7.0-18.0]	12.0[7.0-18.0]	NS
FBG(mmol/L)	4.9[4.6-5.3]	4.8[4.6-5.1]	<0.001
Lipid Metabolic Parameters			
TG(mmol/L)	1.6[1.1-2.5]	0.9[0.7-1.2]	<0.001
TC(mmol/L)	4.2[3.6-5.0]	4.2[3.8-4.7]	NS
HDL-C(mmol/L)	1.0[0.9-1.1]	1.5[1.3-1.7]	<0.001
LDL-C(mmol/L)	2.7(2.2-3.2)	2.4(2.0-2.9)	<0.001
**ART characteristics**			
Total gonadotropin dose (IU)	2781.3[2030.9-3600.0]	2487.5[1762.5-3387.5]	<0.001
No. of oocytes retrieved	12.0[8.0-17.0]	12.0[8.0-17.0]	NS
Insemination method, n (%)			NS
IVF	348(89.7)	2514(88.4)	
ICSI	39(10.1)	304(10.7)	
IVF+ICSI	1(0.3)	26(0.9)	
No. of 2PN embryos	7.0[4.0-11.0]	7.0[4.0-11.0]	NS
No. of available embryos	4.0[3.0-6.0]	4.0[2.0-6.0]	NS
OHSS , n (%)	39(10.1)	309(10.9)	NS
CCPR, n (%)	388(76.5)	2844(74.8)	NS
CLBR, n (%)	270(69.6)	1971(69.3)	NS

Data are presented as mean±SD,the median (inter quartile range, 25th-75th) or frequency (percentage). Abbreviations are the same as in **Table 2**.

**Figure 2:**
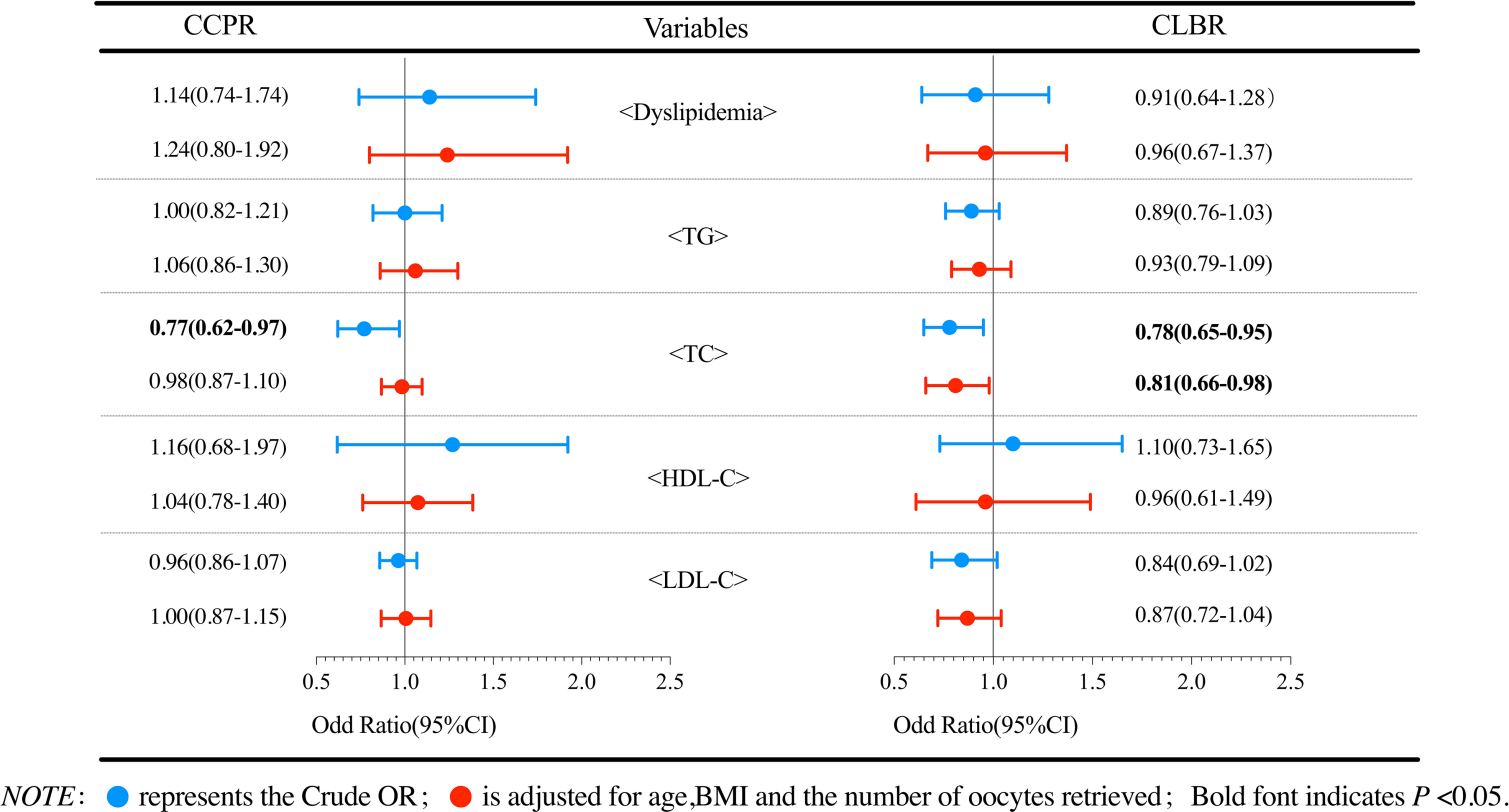
Logistic regression analysis of the effects of dyslipidemia and lipid parameters on cumulative pregnancy outcomes in PCOS patients.

**Figure 3:**
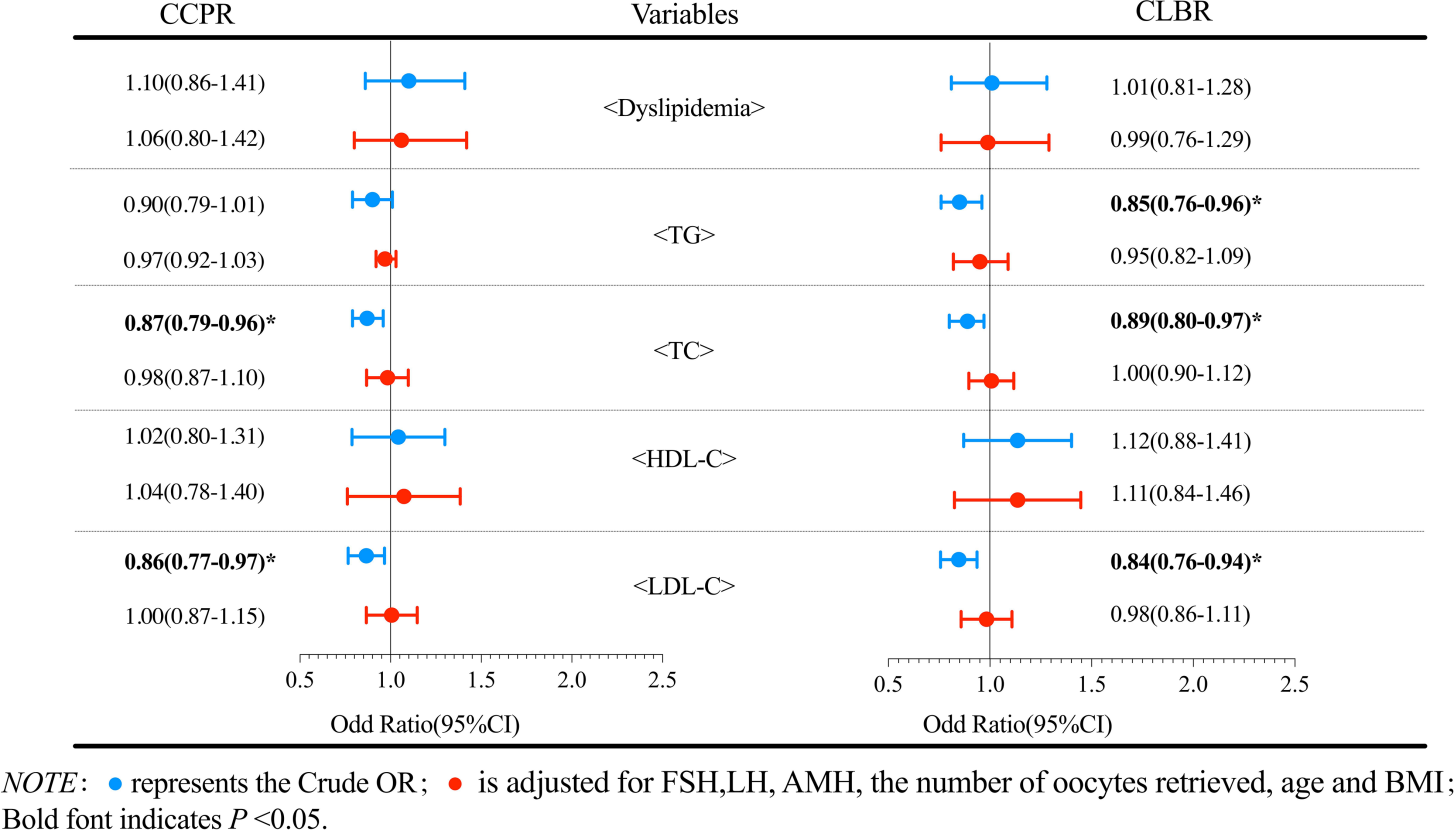
Logistic regression analysis of the effects of dyslipidemia and lipid parameters on cumulative pregnancy outcomes in non-PCOS patients.

Logistic regression analysis was performed to evaluate the effect of dyslipidemia on the cumulative pregnancy outcomes. The results of patients with PCOS are shown in **Figure 2**. Compared with the reference group, the dyslipidemia group had a lower CLBR, but the reductions in ORs were not statistically significant (*P*>0.05). Further analysis of the effects of various lipid metabolic parameters on the cumulative pregnancy outcome revealed that the TC level had an adverse effect on the CCPR and CLBR. After adjusting for confounding factors such as the number of oocytes retrieved, age and BMI, a per unit increase in TC (mmol/L) was found to result in a 19% (95% CI 0.66-0.98) reduction in the CLBR (*P*<0.05). No significant correlations were found in other logistic regression analyses. **Figure 3** shows the analysis results of patients with tubal factor infertility. Similar to patients with PCOS, dyslipidemia also had no statistically significant effect on the cumulative pregnancy outcome in tubal infertility patients. TC [aOR (95% CI): 0.89(0.80-0.97), *P*<0.05], TG [aOR (95% CI): 0.85(0.76-0.96), *P*<0.05], and LDL-C [aOR (95% CI): 0.84(0.76-0.94), *P*<0.05] were negatively correlated with the CLBR, but these associations did not exist after adjustment for confounders, including basal FSH and LH, AMH, the number of oocytes retrieved, age and BMI (*P*>0.05).

In addition, to determine the impact of the degree of TC elevation on the cumulative pregnancy outcomes of PCOS patients, we performed subgroup analysis of various lipid metabolism parameters according to the diagnostic criteria for dyslipidemia. **Table 4** shows the results of the logistic regression for the subgroup analyses. The results showed that the CLBR decreased significantly when TC≥6.22 [aOR (95% CI): 0.26 (0.09-0.78), *P*<0.05], and there was no significant effect on CLBR when TC was defined as marginally increased (*P>*0.05).

**Table 4:** Logistic regression results of subgroup analysis of lipid parameters in PCOS patients.

Lipid Metabolic Parameters		CPBR				CLBR			
		OR (95% CI)	*P* value	aOR (95% CI)	*P* value	OR (95% CI)	*P* value	aOR (95% CI)	*P* value
**TG**									
Normal level	<1.76	Ref (1)				Ref (1)			
Marginal Increase	≥1.67,<2.26	1.09 (0.63-1.90)	0.748	1.13 (0.64-2.00)	0.664	1.08 (0.67-1.73)	0.755	1.11 (0.68-1.80)	0.679
Increase	≥2.26	1.41 (0.79-2.50)	0.249	1.56 (0.86-2.81)	0.142	0.96 (0.62-1.48)	0.837	1.04 (0.66-1.62)	0.879
**TC**									
Normal level	<5.18	Ref (1)				Ref (1)			
Marginal Increase	≥5.18,<6.22	0.99 (0.55-1.76)	0.962	1.02 (0.56-1.84)	0.954	1.09 (0.66-1.81)	0.736	1.11 (0.66-1.87)	0.687
Increase	≥6.22	**0.31 (0.10-0.98)**	**0.047**	0.33 (0.10-1.09)	0.068	**0.26 (0.09-0.75)**	**0.012**	**0.26 (0.09-0.78)**	**0.016**
**HDL-C**									
Normal level	>1.04	Ref (1)				Ref (1)			
Decrease	≦1.04	1.00 (0.62-1.62)	0.989	1.22 (0.71-2.11)	0.480	1.11 (0.71-1.72)	0.647	1.16 (0.74-1.84)	0.521
**LDL-C**									
Normal level	<3.37	Ref (1) Ref (1)				Ref (1)			
Marginal Increase	≥3.37,<4.14	0.74 (0.44-1.25)	0.262	0.77 (0.45-1.32)	0.338	0.82 (0.52-1.31)	0.408	0.85 (0.53-1.36)	0.492
Increase	≥4.14	0.59 (0.17-2.04)	0.402	0.73 (0.20-2.64)	0.633	**0.37 (0.14-0.99)**	**0.047**	0.43 (0.16-1.18)	0.099

aOR (95% CI) are adjusted for age, BMI and the number of oocytes retrieved. Bold font indicates *P* <0.05.

## Discussion

In summary, the principal finding of the study was that TC negatively affected the CLBR after the first ovarian stimulation in PCOS patients. Dyslipidemia caused by elevated TC may result in a decline in the CLBR in PCOS patients.

### PCOS and dyslipidemia

In our study, PCOS patients were more likely to develop dyslipidemia than non-PCOS patients. The metabolic characteristics of PCOS patients were an increased BMI, elevated TG and reduced HDL-C (21). Previous studies have also reported a high prevalence of HDL-C hypolipidemia and hypertriglyceridemia in PCOS patients (22). In addition to known changes in TG and HDL-C, PCOS patients had higher LDL-C and non-HDL-C levels, independent of BMI (14). Abnormal lipid levels have been shown to be independent predictors of atherosclerotic cardiovascular disease (ASCVD) risk (23). Hence, the Androgen Excess and PCOS Society guidelines have recommended that for women with PCOS, a complete lipid profile should be determined to assess their cardiovascular disease risk (24).

Since PCOS is modulated by various endocrine factors, the mechanistic underpinnings of dyslipidemia may be intricate and complex and are still being elucidated. Obesity has been reported to be associated with disturbances in lipid metabolism, including excessive lipolysis that further leads to increased production and secretion of circulating free fatty acids (FFAs), which are considerable substrates for the synthesis of triglycerides. In addition, adipose tissue inflammation caused by obesity can alter the adipokine distribution in adipocytes, thus regulating lipid metabolism through different targets (25, 26). Plasma FFAs also play an important role in obesity and insulin resistance (IR). IR is often used to explain the causes of dyslipidemia of PCOS, where the impaired ability of insulin to inhibit lipolysis leads to increased mobilization of FFAs. One study confirmed that plasma FFA kinetics may be dysregulated due to changes in circulating insulin and glucose in PCOS patients without diabetes, particularly obese patients, which may be related to insulin sensitivity (27). Further research is needed to shed light on the underlying mechanisms of specific patterns of lipid alteration.

### Dyslipidemia and reproductive system disorders

It appears clear that metabolism and endocrinology are tightly connected and reciprocally regulated. Follicle-stimulating hormone and sex hormone-binding globulin were found to predict dyslipidemia in patients with PCOS (21). Our study showed that in the PCOS population, basic endocrine levels were similar in the dyslipidemia group and the normal group. In the non-PCOS population, the dyslipidemia group showed slightly lower levels of FSH and LH. With or without PCOS, the total gonadotropin dose in the dyslipidemia group was significantly higher than that in the normal lipid metabolism group, which was consistent with other studies (18, 19, 28). Some scholars believe that the response sensitivity of gonadotropin decreases due to the disorder of the hypothalamic pituitary gonadal (HPG) axis in patients with dyslipidemia, leading to their need for more gonadotropin to promote follicle growth (29). Since BMI and gonadotropin are risk factors for OHSS, one study found that dyslipidemia may be positively associated with OHSS during the freeze-all embryo cycle (28). Dyslipidemia was observed to lead to reproductive system disorders in animal models of obesity and dyslipidemia induced by a high-fat diet (HFD). A HFD inhibited the formation of primordial follicles and Graafian follicles, impaired oocyte quality, and reduced *in vitro* maturation and fertilization rates (30). Dietary supplementation with soybean oil and cholesterol prior to puberty in rabbits affects the onset of puberty, follicular development, the hormonal response to reproduction, and GnRH stimulation (31). This has yet to be confirmed in additional clinical cohort studies.

### Effects of dyslipidemia on the cumulative pregnancy outcomes after the first ovarian stimulation

In our study, we found that there were no significant differences in the cumulative pregnancy outcomes of PCOS patients after the first ovarian stimulation between the dyslipidemia group and the normal lipid group. Previous studies have shown that dyslipidemia, especially elevated TG levels, in PCOS patients leads to reduced clinical pregnancy rates and live birth rates (32). A recent study targeting PCOS patients undergoing unstimulated natural cycles to rule out the effects of ovarian induction drugs demonstrated that there was no significant correlation between basal lipid metabolism and ART outcomes in PCOS patients (19). We also performed the same analysis in non-PCOS patients, and the results were similar to those in PCOS patients, whereby dyslipidemia had no effect on cumulative pregnancy outcomes. Nevertheless, another study showed that dyslipidemia had an adverse effect on the CLBR in non-PCOS patients, independent of obesity (18). However, we found a negative correlation between total cholesterol and the CLBR in PCOS patients, suggesting that hypercholesterolemia may adversely affect the CLBR. Yang et al. found that TC≥5.20 mmol/L significantly reduced the live birth rates in the first complete cycle of IVF/ICSI in infertile patients (33). Gao et al. used TC>6.11 mmol/L as the diagnostic criterion for hypercholesterolemia, and also found that elevated TC was negatively correlated with the live birth rates of IVF/ICSI in PCOS patients (34). Although diagnostic criteria have not been collaboratively agreed upon, several studies have documented high serum cholesterol as a risk factor for the pregnancy outcomes of IVF/ICSI. Statins are the first-line treatment for hypercholesterolemia, and a retrospective study found that statin therapy significantly improved the pregnancy outcomes after IVF/ICSI in hyperlipidemic patients (35). Cholesterol, an important molecule in mammalian physiology, is crucial for the reproductive system and is involved in the synthesis of steroid hormones. Hypercholesterolemia negatively affects testicular tissue, sperm quality and male fertility hormone levels (36, 37). Elevated plasma cholesterol concentrations may lead to increased oxidative stress in the ovaries, leading to increased follicular atresia (31). Its influence on female fertility and ART outcomes needs to be further clarified. Additionally, more studies are needed in the future to clarify the effect of dyslipidemia on ART outcomes.

### Strengths and limitations

The major advantage of this study was that it investigated the influence of lipid metabolism parameters on the cumulative pregnancy outcomes of PCOS patients and non-PCOS populations separately. It also corrected various factors affecting pregnancy outcomes as much as possible, which allows for more accurate results estimations. Moreover, our study had a large sample size from a relatively homogeneous clinical cohort, which makes our results more reliable. However, there are several limitations to our study. The main limitation of our study is its retrospective nature, which has inherent bias that may affect the results of the study. Second, it is generally recommended that patients with severe dyslipidemia receive lipid-lowering therapy before starting ART in our center, which weakens the generalizability of the results. In addition, we were not able to combine the analysis with the lipid metabolism parameters of males, which should be considered in future studies.

## Conclusion

In conclusion, our findings indicate that TC negatively affected the CLBR after the first ovarian stimulation in PCOS patients. Dyslipidemia caused by elevated TC may result in a decline in the CLBR in PCOS patients. Therefore, a comprehensive assessment of lipid metabolism and scientific management of lipid metabolism, such as weight loss, lipid reduction and lifestyle change, should be carried out. This would not only benefit the long-term health of PCOS patients but may also improve pregnancy outcomes.

## Data availability statement

The raw data supporting the conclusions of this article will be made available by the authors, without undue reservation.

## Ethics statement

The studies involving human participants were reviewed and approved by the Institutional Review Board and Ethics Committee of the First Affiliated Hospital of Zhengzhou University. The patients/participants provided their written informed consent to participate in this study.

## Author contributions

All co-authors were involved in the conception and design of the study. XJ and YG drafted the article. MC and YL were responsible for data collection and verification. XJ and XL performed statistical analysis on the data. All authors critically revised the manuscript for important intellectual content and approved the final manuscript.

## Acknowledgments

We thank all the clinicians and embryologists at the First Affiliated Hospital of Zhengzhou University for their assistance in this study.

## Conflict of interest

The authors state that the study was conducted without any commercial or financial relationships that could be interpreted as a potential conflict of interest.

## Publisher’s note

All claims expressed in this article are solely those of the authors and do not necessarily represent those of their affiliated organizations, or those of the publisher, the editors and the reviewers. Any product that may be evaluated in this article, or claim that may be made by its manufacturer, is not guaranteed or endorsed by the publisher.
